# Black Soldier Fly Full-Fat Larvae Meal as an Alternative to Fish Meal and Fish Oil in Siberian Sturgeon Nutrition: The Effects on Physical Properties of the Feed, Animal Growth Performance, and Feed Acceptance and Utilization

**DOI:** 10.3390/ani10112119

**Published:** 2020-11-15

**Authors:** Mateusz Rawski, Jan Mazurkiewicz, Bartosz Kierończyk, Damian Józefiak

**Affiliations:** 1 Division of Inland Fisheries and Aquaculture, Department of Zoology, Faculty of Veterinary Medicine and Animal Science, Poznań University of Life Sciences, 60-625 Poznań, Poland; mateusz.rawski@up.poznan.pl; 2 Hipromine S.A., 62-023 Robakowo, Poland; damian.jozefiak@hipromine.com; 3 Department of Animal Nutrition, Faculty of Veterinary Medicine and Animal Science, Poznań University of Life Sciences, 60-637 Poznań, Poland; bartosz.kieronczyk@up.poznan.pl

**Keywords:** Siberian sturgeon, black soldier fly, insect meal, growth performance, nitrogen conversion

## Abstract

**Simple Summary:**

Research for alternative protein sources that may replace fish meal and fish oil in fish diets is one of the ongoing tasks for aquaculture. Sturgeons role captive fish production increases due to the rapid decrease in its wild stocks during the 20th century. Insect meals are a novel group of feed materials rich in nutrients that are produced in an environmentally sustainable way. Therefore, the aim of the study was to assess the effects of black soldier fly larvae full-fat meal (BSFL) usage as fish meal and fish oil replacement in Siberian sturgeon diets. The experimentally obtained data showed the possibility of extruded feed production with up to 30% of BSFL and physical parameters suitable for fish feeding. Moreover, feed acceptance increase was observed in treatments containing than 10% and higher shares of BSFL. In the groups whose feed contained 5 to 30% of BSFL in the diet, the growth of experimental fish as well as their feed utilization parameters were improved; however, with no effects on feed digestibility. All presented data make BSFL a suitable nutrient source alternative to fish meal in Siberian sturgeon nutrition.

**Abstract:**

This study provides data on the use of black soldier fly (*Hermetia illucens*) full-fat meal (BSFL) in Siberian sturgeon (*Acipenser baerii*) nutrition, examining pellet physical properties, growth performance, feed acceptance and utilization, apparent protein, and fat digestibility. The study consisted of: feed quality assessment; a growth performance; feed acceptance; digestibility trials. The effect of the use of BSFL as a replacement for fish meal (FM) and fish oil (FO) was investigated. The applied BSFL shares were 5%, 10%, 15%, 20%, 25%, and 30% of the diet, replacing up to 61.3% of FM and allowing us to reduce FO use by up to 95.4% in the case of 30% incorporation. The applied substitution affected feed quality, increasing the expansion rate, and decreasing feed density, sinking speed and water stability. However, body weight gain, specific growth rate, feed, and protein conversion ratios, were improved in groups fed BSFL. Moreover, feed acceptance was increased with treatments containing 10 to 30% BSFL. No effects on nutrients digestibility were observed. The results show that the use of BSFL as an FM and FO replacement may have positive effects on sturgeon growth performance, and BSFL can be developed as a promising alternative feed material.

## 1. Introduction

In the past, fish meal (FM) was the main animal protein source for aquaculture. However, overfishing has increased, threatening many wild fish populations, and together with growing demand resulted in an increase in FM price, and decrease in availability and desirability in aquafeeds [1,2,3]. The predicted shortage of traditional feed materials for aquaculture faces the prospective of rapid development and the growth in the human population leading to an increasing demand for all kinds of resources in terms of feed and food. These include not only basic resources for meeting nutritional requirements but also luxuries, which may be represented by the consumption of wild, expensive, and rare fish species or delicacies such as caviar [4]. What is more, the sustainability should be considered an important factor in the food sector and is perceived as one of the main agents affecting product selection [5,6]. Thus, the sturgeon industry should become more sustainable, independent from wild and environmentally harmful raw materials.

Siberian sturgeon (*Acipenser bareii*) wild populations are estimated to have decreased by 50 to 99.5% during the second half of the 20th century, mainly because of human activity—fishery and environmental degradation. The captive production seems to be the only possible and sustainable way to meet market demands in terms of its meat and caviar. Due to its long and high resource-consuming production cycle, sustainable methods of production, mainly in terms of feed resources, should receive the highest priority in scientific studies. Alternative protein sources for carnivorous fish have been studied for more than 20 years [7]. These protein sources are mainly plant blends. However, the use of plant-derived materials has a negative environmental impact and they cause a number of metabolic disorders in fish due to the presence of antinutritional substances as well as amino acid imbalance [8]. Moreover, their use in sturgeon nutrition is limited because Acipenseridae show higher sensitivity phytoestrogens than other fish species, leading to negative effects on sexual maturation, reproduction, and caviar production [9]. A natural source of nutrients for sturgeons are insects, which are one of the primary prey in juveniles [10]. Currently, insect-derived feed materials are probably the most widely studied topic related to aquaculture nutrition [3,11,12]. The black soldier fly (*Hermetia illucens*) seems to be the most promising species, mainly due to its short rearing period and high biomass gain [13]. Most of the studies in this area are performed on salmonid fish, with little data available for sturgeon [14,15,16,17].

The current state of knowledge provides a wide justification for insect meals use in fish diets; however, the pending goals that should be aimed at in future studies are the levels of replacement application, the most environmentally sustainable and cost-effective forms of insect-derived feed materials for aquaculture.

The studies were planned to verify the use of full-fat black soldier fly larvae meal in Sturgeon nutrition, which is a less popular approach than defatted meals application in fish nutrition. First of all, insect biomass should be considered as the source of protein and fat whose amounts may even be equal in low-processed meals. Most of the literature assumes protein substitution only; thus, the results of defatted meals incorporation to fish diets are most frequently reported [18,19,20,21]. However, the processes of fat extraction and protein purification lower the environmental sustainability and profitability of insect meals application due to high energy consumption, labor, and additional costs. What is more, defatting may decrease the nutritive and functional value of insect meals due to the possibility of amino acid and antimicrobial protein (AMP) degradation in high temperatures and increase in chitin share in the meal, which in high amounts may be considered as an antinutritional factor leading to nutrient digestibility decrease [16]. Moreover, by fat separation, beneficial aspects of fatty acid composition, i.e., high concentrations of lauric acid content, are lowered in insect meals [20].

Despite the wide spectrum of studies on insect meals in fish nutrition, including physiology and microbiota, there are fields that are omitted in most of them. Thus, the presented study aims to answer those basic questions that need to be verified before practical BSFL application in Sturgeon farming. Firstly, the idea of nitrogen to protein conversion factor was reviewed due to previous findings showing that the use of N × 6.25 conversion factor leads to crude protein overestimation [22]. The following stage of the study plan was aimed to answer the question—how do insect meals affect extruded pellet physical properties? These are rarely reported in scientific literature, but highly valuable in terms of practical aquafeed manufacturing, and water quality management—especially in recirculation aquaculture systems [23]. It was followed by in vivo trials aimed to answer the effects of the increasing share of BFSL in the diet on fish growth performance and feed acceptance test—planned to verify the literature information on the negative effects of *Hermetia illucens* meal on feed palatability in Siberian sturgeon [16]. The study plan was closed by another trial aimed at the crude protein and fat digestibility analysis.

## 2. Materials and Methods

### 2.1. Ethics Statement

Studies on live animals were carried out in strict accordance with the recommendations of the National Ethics Commission (Warsaw, Poland). All members of the research staff were trained in animal care, handling, and euthanasia. Fish health and welfare and the environmental conditions in the experimental tanks were checked twice daily by visual observation of animal behaviour and by checking water quality parameters, such as oxygen saturation, temperature, and water flow. At the end of Experiment 1, 14 animals per treatment (2 per tank) were euthanized for tissue sampling for histomorphological analysis. Fish were anaesthetized with an overdose of tricaine methanesulfonate (MS222, 300 mg L^−1^) by prolonged immersion [24]. After sedation, the animals were decapitated according to the American Veterinary Medical Association Guidelines for the Euthanasia of Animals [25]. All remaining animals (294 in Experiment 1, 70 in Experiment 2, and 210 in Experiment 3) were transported to the Experimental Station of Feed Production Technology and Aquaculture in Muchocin for further maintenance. According to Polish law and an EU directive (no 2010/63/EU), the experiments conducted in this study did not require approval from the Local Ethical Committee for Experiments on Animals in Poznań which was confirmed by certificate uploaded during review process.

### 2.2. Insect Meal and Diet Preparation

The BSFL meal was produced at HiProMine S.A., Robakowo, Poland. The BSFL feed was normalized in terms of dry matter (DM) content by the addition of wheat middlings (17%) to a fresh vegetable and fruit mix, consisting of apples (15%), carrots (50%), potatoes (15%), and cabbage (20%) and established at 22% DM. Fresh vegetable and fruit pre-consumer waste was ground (2000 rpm, 55 kW; HPM milling system, Robakowo Poland) to allow passage through a 2-mm screen and offered ad libitum to BSFL. Substrates were not contaminated by any animal products, in accordance with an EC regulation (no 1069/09). At the prepupal stage (10th day of rearing), larvae were harvested, sieved through a 3-mm screen, and washed with water on a drum separator at 90 °C for 10 min (HPM cleaning system, Robakowo, Poland). A batch of BSFL was dried for meal production. The BSFL were dried first at 130 °C for 1 h and then at 80 °C for 23 h until a constant weight was reached using a chamber air flow dryer (HiProMine S.A., Poznań, Poland) to produce BSFL meal and stored at 4 °C before use for feed preparation. Chemical analyses of the dietary ingredients were performed prior to diet formulation in the chemical laboratory of the Department of Animal Nutrition (Poznań University of Life Sciences). For chemical analyses, representative samples of FM, BSFL, and other components were ground to allow passage through a 1.0-mm sieve. Diets and their ingredients, as well as the whole fish, were analysed according to Association of Official Analytical Chemists (AOAC) procedures. Crude protein (N × 6.25) was analysed by the Kjeldahl method after acid digestion (Kjeltec 2300 Auto Analyser, Tecator Hogänas, Sweden). Crude lipids (CLs) were extracted with methyl ether (Soxtec 1043 extraction unit, Tecator, Sweden). DM analysis was performed using the Polish Standard PN-ISO 6496, and ash analysis was performed using the Polish Standard PN–76 R 64795. For crude fibre determination, the Polish Standard PN-EN ISO 6865 was used. All analyses were performed in triplicate according to Józefiak et al. [15,16]. The amino acid content was determined using an AAA–400 automatic amino acid analyzer (Ingos, Prague, Czech Republic) and ninhydrin for post-column derivatization (Ingos, Prague, Czech Republic). Before the analyses, samples were hydrolyzed with 6 N HCl for 24 h at 110 °C [15,16,26]. For TiO_2_ analysis, the samples were prepared in accordance with Myers et al. [27], and the concentration was estimated using the procedure of Short et al. [28] as described by Kierończyk et al. [29]. The analysis of fatty acid composition in BSFL and FM was performed at an accredited laboratory (J.S. Hamilton. S.A. Gdynia, Poland) according to Polish standards PN-EN ISO 12966-1:2015-01, PN-EN ISO 12966-2:2011 and PN-EN ISO12966-4:2015-07. Gross energy of the feeds was determined using an adiabatic bomb calorimeter (KL 12 Mn, Precyzja-Bit PPHU, Bydgoszcz, Poland), Poland standardized with benzoic acid according to Sypniewski et al. [30]. Analysed FM and BSFL compositions are shown in and Table 1 and Table 2, the experimental diets compositions are shown in Table 3.

Analysis of the amino acid composition of proteins was performed to assess the amount of protein and amino acids as well as for conversion factor establishment for BSFL. The BSFL protein content was estimated on the basis of the amino acid content. In further calculations for the diet, the conversion factor (Kp) for BSFL was calculated according to the following formula:

(1) Kp = (total amino acid content/total protein content based on N × 6.25 analysis) × 6.25

This formula is based on the ratio of amino acids to total protein content with N × 6.25 conversion, as previously established by Janssen et al. [22]. The traditional conversion factor of 6.25 was assumed in the case of FM and other materials, which is in agreement with Finke [31] and Gasco et al. [1], who assumed that protein overestimation would occur due to the presence of chitin in insect biomass. The main aim of the calculation was to determine to what extent this most frequently used conversion factor (6.25) is based on an overestimation of the true protein content in BSFL.

Example of a calculation based on the analysis of BSFL used for the presented experiment:

(2) BSFL Kp = (349.8 g/427.1 g) × 6.25=5.12

where 349.8 g is the sum of amino acids in 1000 g of BSFL DM.

427.1 g is the total protein content in 1000 g of BSFL calculated on an N × 6.25 basis;

6.25 is the traditionally used Kp;

5.12 is the Kp calculated for BSFL.

In total, seven diets were calculated: control (CON), containing 26.1% FM and 0% BSFL; H5, with 23.4% FM and 5% BSFL; H10, with 20.8% FM and 10% BSFL; H15, with 18.1% FM and 15% BSFL; H20, with 15.5% FM and 20% BSFL; H25, with 12.8% FM and 25% BSFL; H30, with 10.1% FM and 30% BSFL. Feeds were prepared by extrusion processing with a single-screw warm extruder (Metalchem S-60 Gliwice, Poland) at the Experimental Station of Feed Production Technology and Aquaculture in Muchocin (Poznań University of Life Sciences). The processing conditions were as follows: 90 °C cylinder temperature in the zone of increasing pressure, 110 °C cylinder temperature in the zone of high pressure, 120 °C head temperature, 52 rpm screw speed and 3 mm nozzle diameter. In post-production, fish oil was added by vacuum coating (Rollermac BA 15 FR aut. Pomati Group S.R.L, Codogno, Italy).

### 2.3. Feed Physical Properties

All feed analyses were performed at a temperature of 21 °C and under 60% air-controlled humidity (Toyotomi, YD-C312, Nagoya, Japan). The expansion ratio (ER) was measured as the ratio of pellet diameter to extrusion matrix perforation diameter. The analysis was performed on 20 pellets from each batch using an electronic calliper (Yato, YT-7201, Shanghai, China) with accuracy up to 0.01 mm. The expansion was calculated according to the formula established by Khater et al. [32] and previously used for extruded pellets containing black soldier fly meal by Irungu et al. [33] as well as by Das et al. [34] for other animal derived feed materials use assessment.

(3) ER (%) = ((pellet diameter (mm)/matrix diameter (mm)) × 100

Pellet bulk density (PD) analysis was performed using a modified procedure described by Irungu et al. [33]. A total of ten randomly sampled replications of 1000 mL of feed were weighed (W), and the density was expressed as g/dm^3^.

(4) $$\mathrm{PD}\;\left(g/{\mathrm{dm}}^{3}\right)\;=\;\mathrm{sample}\;\mathrm{weight}/{\mathrm{dm}}^{3}$$

Sinking velocity (SV) was measured according to a modified version of the protocol of Das et al. [34]. A 105-cm-high water column was filled with 100 cm of water. A total of twenty randomly selected pellets per feed batch were inserted into the water individually, and the time taken to reach the bottom was measured with an accuracy of 0.1 s.

(5) SV (s/100 cm) = sinking time (s)/ water column height (cm)

Water stability (WS) was measured as the % of feed mass that disintegrated during 10 min in water according to the procedures of Das et al. [34] and Umar et al. [35], which were modified for better replication of practical sturgeon feeding conditions. For each feed, 100 g (initial sample weight) was placed in a flask, and 500 mL of distilled water was added and placed on a horizontal shaker. After 10 min, feeds were filtered through a 1-mm strainer on paper filters. After water filtration, the residues were dried at 50 °C for 24 h and weighed (final sample weight) to calculate the disintegrated feed proportion. Each feed was analysed in 10 replications.

(6) WS (%) = ((final sample weight − initial sample weight)/initial sample weight) × 100

For better assessment of feed performance in water, the increase in volume (VI) was also assessed. A volume of 25 mL (initial volume) of feed was placed in a volumetric flask, and the flask was filled with 100 mL of water. After 10 min, the volume of the feed (final volume) was measured, and the expansion in water was calculated.

(7) VI (%) = ((final volume − initial volume)/initial volume) × 100

### 2.4. Animal Experiments

#### 2.4.1. Experiment 1: Growth Performance and Feed Utilization

The growth trial was carried out using 392 Siberian sturgeon fingerlings (mean body weight 14.4 ± 3.0 g). The fingerlings were obtained from the Experimental Station of Feed Production Technology and Aquaculture in Muchocin (Poznań University of Life Sciences), and after 7 days of acclimation, they were randomly distributed into 49 rectangular fibreglass tanks (60 dm^3^ capacity). A total of 56 fish were used per treatment, with 8 fish per tank (mean body weight 14.4 ± 2.0 g). The experiment was conducted over 50 days. The study design included seven treatments, with seven replications (tanks) per treatment.

The experimental unit was arranged as a recirculation system for the Division of Inland Fisheries and Aquaculture (Poznań University of Life Sciences); the total capacity was 10.6 m^3^, with 66 experimental tanks, refreshments daily at the level up to of 5% with tap water. The flow-through from each tank was at a constant level of 3 L/minute, which allowed replacement of the total volume of water three times per hour. During the entire experimental period (for all 3 tests), the average daily water temperature (Table 2) was 20.3 °C; the dissolved oxygen level was 8.42 mg O^2^/dm^3^ (WTW Multi Line P4 with an optical oxygen sensor (WTW, FDO 924, 99, Weilheim, Germany); the water pH was 8.81 (WTW Multi Line P3 pH meter, WTW, Weilheim, Germany); the conductivity was 856 µS (HM Digital Inc., EC-3, Redondo Beach, CA, USA); the ammonia content was 0.06 mg/dm^3^; the NO_2_ content was 0.12 mg/dm^3^; the NO_3_ content was 12.7 mg/dm^3^ (Merck MColortest cat no. 1144230002, 1144240001, 111170.0001, respectively, Merck Darmstadt, Germany).

The photoperiod was maintained at 16:8 (light:dark) during the entire experiment, with light provided from 07:00 to 23:00. Fish were fed every day with automatic band feeders for 12 h per day (08:00 to 20:00). For feed intake control and accuracy, fish were weighed every 10 days, and the feeding rate was corrected according to body weight and water temperature as described by Hung et al. [36]. Feed consumption was controlled 5 times daily during the feeding period. If any uneaten feed was observed, it was siphoned, dried, and weighed to increase the accuracy of feed intake measurements and feed supply correction for the following day. The effects of the diets were evaluated on the basis of fish biomass, the amount of feed consumed, diet utilization, and dissection of internal organs. Equations recommended by Hardy and Barrows [37] were used to calculate growth performance. The calculated parameters included final body weight (FBW), body weight gain (BWG), specific growth rate (SGR), percent of body weight gain (PWG), feed intake (FI), feed conversion ratio (FCR), protein efficiency ratio (PER), and survival rate (SR). The formulas used in the experiment are presented below:

(8) FBW (g) = fish biomass in the tank (g)/number of fish in the tank

(9) BWG (g) = final body weight (g) − initial body weight (g)

(10) SGR (%/day) = (ln final body weight − ln initial body weight)/number of feeding days) × 100

(11) PWG (%) = (final body weight (g) − initial body weight (g)/initial body weight (g)) × 100

(12) FI (g) = applied feed (g) − uneaten feed (g)

(13) FCR = feed intake (g)/body weight gain (g)

(14) PER = (body weight gain (g)/(feed intake (g) × protein level in the diet (%)))

(15) SR (%) = (final number of live fish/initial number of live fish) × 100

#### 2.4.2. Experiment 2: Feed Acceptance Test

The observations made during Experiment 1 showed a non-significant trend in FI, which increased with BSFL share in the diet. To confirm this finding, the feed acceptance test was performed on 210 Siberian sturgeons (mean body weight 50 ± 5.0 g). The fish were obtained from the same spawning material as in Experiment 1. The animals were randomly distributed into 21 rectangular fiberglass tanks (60 dm^3^ capacity, 3 tanks per treatment). A total of 30 fish were used per treatment, with 10 fish per tank. The fish were acclimated to experimental conditions for 7 days and fed commercial feed (Aller Bronze, Aller Aqua Polska sp. z o.o. Nożynko, Poland). The photoperiod was maintained at 16:8 (light:dark) during the entire experiment, with light provided from 07:00 to 23:00. After the initial period, fish were fed experimental feeds 3 times a day for 3 days at 08:00, 13:00, and 18:00. Hand-feeding of each experimental feed lasted for 10 min, until visible satiation was attained. If any feed residues remained, they were siphoned, dried, and weighed for actual feed intake calculations as described for Experiment 1. The results are given in relation to fish body weight according to the calculation shown below:

(16) Feed acceptance (% of body weight/feeding session) = (feed intake (g/session)/biomass of fish in the tank) × 100

After the feed acceptance test, 140 fish were transferred to metabolic tanks for the digestibility test as per the 4R (Reduce, Reuse, Recycle, and Restore) strategy for animal experimentation and for performing further trials on the effects of BSFL in Siberian sturgeon nutrition.

#### 2.4.3. Experiment 3: Nutrient Digestibility Coefficient Assessment

The digestibility test was performed using 140 fish randomly distributed to 14 cylindrical fiberglass tanks (60 dm^3^ of fish living space and 18 dm^3^ for feces sedimentation). Ten fish/tank and 2 tanks per treatment were used to determine the apparent digestibility coefficients of nutrients in experimental diets for Siberian sturgeon. Metabolic tanks were used as per the design of Allan et al. [38] with modifications. The tanks were equipped at the conical bottom with a separation sieve and a two-valve separation and drainage system. The photoperiod was maintained at 17:7 (light:dark) during the entire experiment, with light provided from 07:00 to 00:00. The digestibility test lasted 30 days, including 15 days of adaptation, and 15 days of fecal sampling. Animals were fed with the same diets used in the growth performance trials with the addition of 0.3% titanium oxide (TiO_2_) as a digestibility marker. The feed was provided at 1% fish weight per day for 6 h (from 7:30 to 13:30) using automatic belt feeders. After feeding, the residues of uneaten feed were removed by siphoning with the bottom valves. The accumulated feces were obtained by opening the bottom separation system and collecting feces for filtration on cellulose filters. The obtained material was frozen at −20 °C for further analysis. Fecal collection was conducted 3 times per day at 07:00, 18:00, and 23:00. The experiment was terminated on the 30th day to maintain the highest standards of animal welfare, to avoid tank overcrowding, based on the higher than expected growth of sturgeons receiving BSFL treatment. Due to analytical needs and the high water content, the collected feces were pooled into one sample/treatment for further analysis performed according to the methods described above for feed ingredients and feeds.

The coefficients of nutrient digestibility of CP and EE in sturgeons were calculated relative to the proportion of TiO_2_ (dietary marker) to determine the nutrient content in feed or feces according to Kaczmarek et al. [39]. The following equation was used according to the modified formula described by Kierończyk et al. [29].

Digestibility of nutrient = 1 − ((TiO_2_ (g/kg) in the diet/TiO_2_ (g/kg) in the faeces) × (nutrient (g/kg) in the faeces/nutrient (g/kg) in the diet)). (17)

### 2.5. Statistical Analysis

In the case of feed physical properties, n/treatment is given in the methodological descriptions for each parameter. For growth performance, feed utilization, and acceptance trials, the experimental unit was the tank n/treatment (n/treatment = 7 in Experiment 1; n/treatment = 3 in Experiment 2. In the case of digestibility coefficients, sample pooling was necessary; thus, n/treatment = 1 (2 samples pooled per treatment) represent faeces collected from 10 fish per tank—20 fish per treatment.

All obtained data for feed physical properties and acceptance, growth performance, and feed utilization analysis were tested for normal distribution using the Kolmogorov–Smirnov test. Analysis of variance homogeneity was conducted using Bartlett’s test. The significance of differences between groups was determined by Duncan’s multiple range test at a significance level of *p* ≤ 0.05. The calculations were performed using SAS 9.4 software. The following general model was used:

(18) $$Y_{i}\;=\;\mu \;+\;\alpha_{i}\;+\;\delta_{\mathrm{ij}}$$

where Y_i_ was the observed dependent variable; μ was the overall mean; α_i_ was the effect of insect inclusion; δ_ij_ was the random error according to Rawski et al. [40].

For analysis of the data from the digestibility trial by linear regression and correlation, models were used.

## 3. Results

### 3.1. Raw Materials

The chemical compositions of BSFL and FM are given in Table 1. BSFL was characterized by a lower protein and crude ash content than that of FM; however, the crude fat, fibre, and nitrogen-free extract levels were higher. On the basis of amino acid analysis, the Kp for BSFL was established as 5.12, which is 0.82 of 6.25. The amino acid profiles of BSFL and FM were similar in terms of lysine, glutamine, serine, glycine, histidine, arginine, tryptophan, isoleucine, and leucine levels. Higher levels of valine, tyrosine, proline, alanine, and phenylalanine, and markedly lower levels of aspartic acid and cystine were observed in BSFL than in FM. The BSFL and FM showed different fatty acid compositions (Table 2), in BSFL dominant fatty acids were C12:0 (lauric acid), C18:1 n9 (oleic acid), C18:2 n6 (linoleic acid) and C14:0 (myristic acid) while in fish meal the highest levels of C22:6 n3 (docosahexanoic acid), C18:1 n9 (oleic acid) and C20:5 n3 (eicosapentaenoic acid) were observed.

### 3.2. Feed Composition and Physical Properties

Feed production using the extrusion method was successfully performed for all treatments. No effects on extrusion, drying, or oil coating processes were observed. All experimental diets are reported in Table 3 in terms of composition and nutritive value. The DM (93.45–93.60%), crude protein (48.55–50.74%), crude fat (9.21–10.24%), crude ash (8.09–8.23%) as well as gross energy (20.39–20.44 MJ/kg) levels were comparable among treatments. The levels of FM replacement reached in the experiment were 0%, 10.3%, 20.3%, 30.7%, 40.6%, 50.9%, and 61.3%. The use of BSFL reduced the use of fish oil (FO) by 0%, 15.4%, 32.3%, 47.7%, 61.3%, 78.5%, and 95.4%, both of which are presented in Table 3. The increasing proportion of BSFL decreased the use of limestone from 1.8 to 1% and led to the need for phosphate as a Ca and P source (Table 3). The use of BSFL affected feed physical properties, including pellet diameter and expansion rate (*p* = 0.0017), pellet density, and SV (*p* < 0.0001). Pellet diameter and expansion rates were increased for the H15, H20, H25, and H30 diets. There were no differences among the H5, H10, and H15 diets. PD was the highest in the CON and H5 treatments, and a further increase in the BSFL proportion caused a significant decrease in density. The SV was decreased by H20 and H25, and the lowest value was observed for H30. WS was worsened by the use of BSFL meal in the H25 and H30 groups. The loss of structure (increase in volume) after 10 min in water was higher in the H15, H20, and H25 treatments, and the highest value was recorded in the H30 treatment (Table 4).

### 3.3. Growth Performance, Feed Utilization, and Acceptance

No mortality or illness symptoms were observed during the entire trial among all the treatments, and the survival rate was 100% in the whole experimental period (Table 5). Body weight and body weight gain specific growth rate, as well as relative growth rate, were significantly increased by the use of BSFL meal. The lowest values were observed in the control treatment, and the H5 treatment results were significantly lower than those for H10, H15, H20, H25, and H30. There were no differences in feed intake among the treatments. The FCR as well as the PER were improved by the use of insects; however, among the treatments, the FCR was highest in the H5 treatment, and there were no differences among the higher BSFL doses. In the case of PER, the lowest value was observed in the control treatment, with significant improvement in H5, while H10, H15, H20, H25, and H30 showed higher values.

### 3.4. Digestibility Coefficients

**Figure 1:**
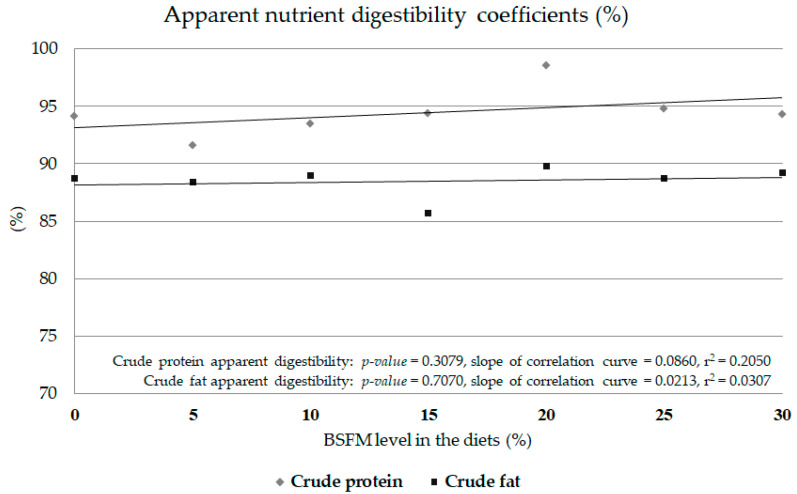
Apparent nutrient digestibility coefficients (%) in Siberian sturgeon fed experimental diets containing black soldier fly full-fat meal (Experiment 3). Points on the horizontal axis represent the level of BSFL inclusion and treatments. 0: CON, control feed; 5: H5, experimental feed with 5% BSFL; 10: H10, experimental feed with 10% BSFL; 15: H15, experimental feed with 15% BSFL; 20: H20, experimental feed with 20% BSFL; 25: H25, experimental feed with 25% BSFL; 30: H30, experimental feed with 30% BSFL.

In terms of the total tract digestibility of crude protein and fat, there was no effect of insect meal inclusion in the diet (Figure 1). There were no significant correlations among the dose of BSFL and crude protein digestibility (*p* = 0.3079) or crude fat digestibility (*p* = 0.7070). The slopes of the correlation curve were low for both parameters: crude protein digestibility (0.0860) and crude fat digestibility (0.0213). In both cases, the r^2^ shows a lack of a BSFL share in the diet effect on digestibility coefficients (0.0307 for crude fat and 0.2050 for crude protein).

## 4. Discussion

The proximate composition of components used in the study is in agreement with values reported in the scientific literature as well as those previously used in our trials [3,15,16,41,42,43]. Similar to the results of Irungu et al. [33], who replaced shrimp meal with insect meals and observed lower crude protein content and higher crude fat and crude fibre levels in BSFL than in FM and other traditionally used protein sources. The above-mentioned differences between FM and BSFL affected the maximal level of BSFL, which may be incorporated into the diet. With over 30% share of BSFL, the calculated potential environmental sustainability of the diet, as well as the extrusion process and feed properties may be impaired. The current study confirmed the findings of Janssen et al. [22] in terms of the crude protein conversion factor (Kp), which was previously assumed for *H. illucens* biomass to range from 4.76 to 5.60, depending on crude protein quality and purification. The Kp factor for the material used in the present experiment was calculated as 5.12 on the basis of amino acid content, which means that in the N × 6.25 calculation, 18% overestimation of crude protein occurred. However, in whole-feed composition analysis, it is impossible to apply the above-mentioned Kp due to the lack of possibility of differentiation of nitrogen of insect origin from that of other sources. Thus, in the nitrogen-based analysis of the feeds, a slight increase in the level of crude protein was observed with increasing proportions of BSFL in the diet. This was probably caused by the presence of non-amino-acid nitrogen, which is bound in chitin, even though the diets were balanced on the basis of the modified Kp as an isoprotein. Thus, we suggest that when insect-containing practical feed compositions are calculated, the application of Kp modification, or feed balancing on the basis of amino acids content should be applied to meet fish nutritional needs. However, the increase in protein content observed among the treatments seems to not be relevant for the observed growth performance improvement based on the high flexibility of young sturgeon in this area [44]. Moreover, if the differences in detected protein level among the studies would be biologically significant, the expectation should be a decrease in the growth performance progressing above the CP level over 45% [45]. The use of increasing amounts of BSFL has led to a decrease in limestone use and an increase in phosphate use for maintenance of the dietary Ca:P ratio (1.67–1.85:1) and phosphorus content (0.81%) at optimal levels [46]. Thus, in feed calculation and manufacturing, when insect meals are used, attention should be paid not only to chitin-associated protein estimation issues but also to the mineral balance of the diet. Moreover, in the present situation, phosphorus retention and excretion to the environment may be considered important elements for further studies on insect-derived feeds in terms of their environmental sustainability as practical low-pollution feeds [47]. It is worth underlining that the use of full-fat BSFL allowed for high fish meal replacement (61%), but even higher (95%) replacement of fish oil, which shows the additional value of this form of insect meals in environmentally sustainable diets for aquaculture.

Crude fat is the second nutrient observed in the insect biomass, and it should be considered in terms of feed processing by extrusion and fish metabolism. In the literature, as well as in the context of aquafeed production, it has been widely discussed that oils added to the feed before the cooking extrusion process decrease the quality of pellets and the expansion of the feed [48,49]. In the present study, increases in pellet diameter and expansion rate were observed together with the increasing proportions of insects and the amount of crude fat present in the extruded materials. Despite the general assumption, this was previously observed with selected plant oil additions [50]. Fat is considered a factor that may decrease expansion due to decreased input of mechanical and thermal energy received by feed, as well as starch gelatinization due to binding and coating of starch particles [48]. However, Irungu et al. [33] did not observe the effect of the insect meal source and inclusion level when *H. illucens* and *Acheta domesticus* full-fat meals were used, while, contrary to the present results, Weththasinghe et al. [49] observed a decrease in pellet expansion due to *H. illucens* usage. The obtained results may suggest that when full-fat *H. illucens* meal is used, the oil is much better dispersed in the feed than in when it is separately added as a liquid prior to extrusion. This causes equal distribution and mixing during extrusion because fat is provided in the whole volume of insect meal. This also causes extrusion to become an oil extraction process; thus, the lipids become oils that are dispensed in very small drops (<1 µm) and start to provide strong lubrication, which decreases the amount of mechanical energy needed for extrusion and increase the proportion of oil, which may be used for the cooking process [51]. The fats provided in the above-mentioned form may have lower coating effects and do not affect starch gelatinization by smearing the biopolymer particle surface. Moreover, when oil extraction from the full-fat meal becomes a part of extrusion, it is highly probable that starch gelatinization and the formation of a highly expandable foam structure partially occur before the fat is extracted from the raw material particles. It was previously widely shown that extrusion effects are dependent in not only the amount but also the source of fat and starch [48]. It was also suggested that the sensitivity of starch to the worsening of extrusion effects due to the presence of fat depends on the source. Thus, the effects of insect oil may differ among studies due to variability in the starch source and physical properties of fat, i.e., melting point or viscosity [48]. The observed higher ER is in agreement with the decrease in SV and PD, which can be explained by increased pore formation in the pellet structure. In previous studies on insect fat application, Dumas et al. [22] observed increased floatability together with a decreased bulk density of the feed. However, both studies are at odds with the results of Irungu et al. [33], who observed a decrease in floatability dependent on BSFL incorporation. Thus, this finding may support the above-mentioned hypothesis that not only the amount but also the form of fat included in the extrusion process may affect the effects of its presence. However, the results of water stability tests, including the VI after soaking and WS, support the presence of the above-mentioned coating effect of fat and partial starch gelatinization blockage, resulting in worsened pellet stability, despite a lack of negative influence on its expansion. The results show that the main changes in the properties of the feed occur between 10 and 20% BSFL proportion, and the highest decrease in pellet WS was observed between the H15 and H20 treatments, which suggests that from a technological perspective, it may be the top advised the limits for insect incorporation into extruded aquafeeds, or alternative processing technologies should be developed.

Nutritional studies on the use of insect meal for Siberian sturgeon are a novelty. In the current literature, there are three reports describing the application of three insect species in the diet of this species [15,16,17]. However, none of these studies presented a feed physical properties analysis or showed clearly positive results of BSFL usage.

The feed acceptance test results suggest that insect-derived materials may be strong attractants for animals. This confirms the results of Murofushi et al. [52], Kasumayan et al. [53] and Kierończyk et al. [54]. Thus, this aspect should be considered as an additional advantage of insect meal use and may be explained by the presence of aromatic compounds [54]. It was previously shown that in the case of defatted *H. illucens* meal, a decrease in feed intake may be observed with the presence of this material in the Siberian sturgeon diet at 18.5% [16]. The authors suggested that increasing feed intolerance with insect meal occurred with the refusal of consumption when *H. illucens* defatted meal was provided. It was explained as the effect of increasing levels of chitin. However, in the cited study, it was the only source of animal protein, at a level of 75% of the diet, which may suggest a negative effect of a single protein source in the diet or adverse effects of the defatting process [16]. No similar negative effect was observed by Józefiak et al. [15] with the incorporation of 20% full-fat *H. illucens* meal. Studies on sturgeon olfaction underline its strong role and connection with protein composition in feeding behaviour [55]. Free amino acids are important stimuli for sturgeon chemoreception. Alanine and valine, which are present at higher concentrations in BSFL than in FM, are strong extraoral and taste stimulants in Siberian sturgeon [55]. In the case of the closely related stellate sturgeon (*Acipenser stellatus*), it was shown that in the comparison of 19 ingredients that contained natural and artificial feed aromas, the most attractive in terms of smell and taste were Chironomidae insect larvae [53]. Insect haemolymph is known as rich in free amino acids, moreover, low molecular weight of *Hermetia illucens* proteins range mainly between 20 to 245 kDa, which may suggest the high share of low molecular weight peptides and free amino acids—which are considered as fish attractants in BSFL [56,57,58]. However, more experiments on insect-derived materials that are attractive for fish are required for future application to explain the role of insect protein, fat and chitin in feed acceptance.

In the present study, there were no significant differences among treatments in terms of feed intake, which suggests that all of the feeds could be provided to fish as whole daily rations without consumption refuse. However, due to a restricted feeding regime in the growth performance trial its results may not be compared with the provided acceptance test in which fish were fed ad libitum. For all other growth performance and feed utilization parameters, including BW, BWG, SGR, PWG, FCR, and PER, an improvement was observed as a consequence of increasing BSFL incorporation into the diets. All the above-mentioned results are in agreement with the digestibility coefficients, as there was no worsening of protein and fat digestibility as the levels of BSFL inclusion increased. This suggests that the physical properties, palatability and nutritive value of experimental diets are satisfactory for Siberian sturgeon nutrition.

The observed improvements in growth performance may be interpreted mainly by the functional role of insect protein – containing AMP and fat composition—with high share of lauric acid as well as chitin prebiotic properties which are widely discussed in the literature [59,60,61,62]. AMP presence positively affects the microbiome, while the presence of high concentration of lauric acid (21 g/100 g of crude fat) may also play a key role in fish health—immune system stimulation, and microbiome stabilization also [62]. Moreover, considering the use of full-fat BSFL, and available sources of scientific knowledge on insect fat use [21,59,60], there is no confirmed data on negative effects of insect fat – even in total replacement cases. What is more positive effects of *Hermetia illucens* oil use in fish diets on fat metabolism in the case of soybean oil replacement. Li et al. [60] observed a decrease in intraperitoneal fat deposition, adipocyte size, increased n-3 polyunsaturated fatty acids content as well as up-regulation of the expression of lipid hydrolysis mediating PPARα gene. The obtained results were comparable with the study of Józefiak et al. [15,16], who did not observe adverse effects of *H. illucens* full-fat meal incorporation. However, they are at odds with the study of Caimi et al. [17], in which growth performance and feed utilization parameters, as well as digestibility coefficients, were impaired by the use of defatted black soldier fly meal. The lack of differences in nutrient digestibility coefficients may be interpreted as the effects of a lower level of chitin in full-fat meals in comparison with defatted ones. Chitin in fish nutrition—depending on the dose—is considered as a prebiotic substance or antinutritive compound which may decrease feed palatability, nutrient digestibility, and growth performance [62,63,64,65,66]. It was considered an immunostimulant in the case of yellow catfish (*Pelteobagrus fulvidraco*) [67]. It should also be emphasized that chitin is the most commonly ingested carbohydrate in sturgeon diets, which in the early stages of growth tolerate even insect-based monodiets thus they may show higher tolerance than other fish taxa [68]. The occurrence of physiological changes in sturgeon, i.e., pathogen decrease or gastrointestinal tract histomorphological alteration, as observed by Józefiak et al. [16] are also possible.

The consideration of the full mode of action observed in the presented study should be started form the level of diet composition, which was based on the modified nitrogen to protein conversion factor to ensure the balance of the diets. Experimentally produced feeds were well accepted by fish, which was confirmed during acceptance test, and resulted in lack of differences in feed intake during the growth performance experiment. BSFL is rich in lauric acid, and AMP improved growth performance and feed utilization. Due to its full-fat form, the negative effects of chitin presence was not observed in terms of digestibility coefficients, which allowed fish to use their growth potential.

The observed results, as well as the idea of insect meals use, should be considered in comparison with studies on insect fat use in fish nutrition also. The high amounts of crude fat in insect biomass should not be neglected nor omitted in the general trend of insect use as the source of protein. To keep high sustainability, the insect products should be used in low processed form. Full-fat insect meals seem to be an optimal compromise between fresh biomass and cost and resource-consuming protein and oil separation-purification process. Moreover, it is unknown if physical and chemical factors used in defatting may negatively affect protein biological value, as well as health beneficial antimicrobial peptides, chitin, and fatty acid composition.

Thus, the use of full-fat insect meals may be suggested as the most efficient and environmentally sustainable method of providing nutrients for fish with no adverse effects of high chitin levels in defatted products and allows to fully use the high potential of *Hermetia illucens* biomass.

## 5. Conclusions

In summary, all the above-mentioned results show that BSFL use in Siberian sturgeon diets may be assessed on the basis of feed property analysis and in vivo experiments, including by examining feed acceptance, growth performance, feed utilization, and nutrient digestibility. The empirical amino acid-based conversion factor (Kp = 5.12) for *H. illucens* protein was established. The use of BSFL did not impair feed formulation, production, or physical parameters, and BSFL use at a level of 30% inclusion led to 61% FM and 95% FO replacement. Moreover, insect meals may act as natural feed intake stimulants due to the nutritional biology of Acipenseridae. The results show that sturgeons tolerate insect meal inclusion in their diets, and insect meal may act as a growth stimulant. The feed utilization as well as nutrient digestibility parameters suggest that sturgeons may respond much better than other fish to this group of feed ingredients. The full-fat meals or insect oils may be promising alternative feed sources for Siberian sturgeon.

## Figures and Tables

**Table 1:** Nutritive value and amino acid profile of Black soldier fly full-fat larvae meal (BSFL) and fish meal (FM) used in the experiments.

Nutrient-	BSFL	FM
	g/1000 g of dry matter	
Crude protein	350	618
Crude fat	298	165
Crude fibre	79.0	0
Crude ash	53.0	175
Nitrogen free extract	221	42.0
Amino acid	g/100 g of crude protein	
Aspartic acid	7.30	9.40
Glutamic acid	13.1	14.5
Serine	4.88	4.17
Glycine	6.15	6.41
Histidine	3.25	2.09
Arginine	5.47	6.07
Threonine	4.43	4.10
Alanine	8.21	6.87
Proline	6.68	4.28
Tyrosine	6.71	3.00
Valine	6.79	5.79
Methionine	2.12	2.53
Cystine	0.76	9.59
Isoleucine	4.73	4.24
Leucine	7.83	7.48
Phenylalanine	7.76	3.07
Lysine	6.82	6.63

**Table 2:** Fatty acid profile of Black soldier fly larvae full-fat meal (BSFL) and fish meal (FM).

Fatty Acid	BSFL	FM
	g/100 g of crude fat	
C12:0 (lauric acid)	21.00	<0.1
C13:0 (tridecylic acid)	<0.1	<0.1
C14:0 (myristic acid)	3.70	5.30
C15:0 (pentadecanoic acid)	<0.1	1.10
C16:0 (palmitic acid)	5.30	5.30
C17:0 (margaric acid)	<0.1	1.40
C18:0 (stearic acid)	0.80	4.40
C20:0 (arachidic acid)	<0.1	0.20
C21:0 (heneicosanoic acid)	<0.1	0.20
C22:0 (behenic acid)	<0.1	0.20
C23:0 (tricosylic acid)	<0.1	0.10
C24:0 (lignoceric acid)	<0.1	0.20
C14:1 (myristoleic acid)	<0.1	0.20
C15:1 (ginkgolic acid)	<0.1	<0.1
C16:1 n7 (palmitoleic acid)	1.60	5.30
C17:1 (margaroleic acid)	<0.1	<0.1
C18:1 n9 (trans-elaidic acid)	<0.1	0.20
C18:1 n9 (oleic acid)	6.10	13.00
C18:1 n7 (vaccenic acid)	0.20	<0.1
C20:1 n9 (eicosenoic acid)	0.10	1.50
C22:1 n11 (gadoleic acid)	<0.1	<0.1
C22:1 n9 (erucic acid)	<0.1	0.20
C24:1 n9 (nervonic acid)	<0.1	<0.1
C16:2 n4 (hexadecadienoic acid)	<0.1	<.01
C16:3 n4 (hexadecatrienoic acid)	<0.1	<0.1
C18:2 n6 (trans-linolelaidic acid)	<0.1	0.20
C18:2 n6 (linoleic acid)	3.80	1.70
C18:3 n6 (γ-linolenic acid)	<0.1	0.10
C18:3 n4 (octadecatrienoic acid)	<0.1	<0.1
C18:3 n3 (α-linolenic acid)	0.50	1.00
C18:4 n3 (stearidonic acid)	<0.1	<0.1
C20:2 n6 (eicosadienoic acid)	<0.1	0.30
C20:3 n6 (dihomo-γ-linolenic acid)	<0.1	<0.1
C20:3 n3 (eicosatrienoic acid)	<0.1	<0.1
C20:4 n6 (arachidonic acid)	<0.1	1.20
C20:4 n3 (eicosatetraenoic acid)	<0.1	<0.1
C20:5 n3 (eicosapentaenoic acid)	0.20	7.90
C22:2 n6 (docosadienoic acid)	<0.1	1.10
C22:5 n3 (docosapentaenoic acid)	<0.1	<0.1
C22:6 n3 (docosahexaenoic acid)	<0.1	18.30

**Table 3:** Compositions and proximate chemical analysis of experimental diets.

Treatment	CON	H5	H10	H15	H20	H25	H30
	Diet composition (g/1000 g)						
Fish meal	261	234	208	181	155	128	101
Red blood cells	100	100	100	100	100	100	100
BSFL	0	50	100	150	200	250	300
Soy protein isolate	100	100	100	100	100	100	100
Wheat gluten	150	150	150	150	150	150	150
Wheat meal	145	130	117	104	89	76	63
Maltodextrin	130	130	130	130	130	130	130
Fish oil	65	55	44	34	24	14	3
Lecithin	10	10	10	10	10	10	10
Premix ^1^	15	15	15	15	15	15	15
Vitamin premix ^2^	1	1	1	1	1	1	1
Choline chloride	2	2	2	2	2	2	2
Limestone	18	18	16	14	3	11	10
Phosphate 1-Ca	0	2	4	6	8	10	12
TiO_2_	3	3	3	3	3	3	3
Total	1000	1000	1000	1000	1000	1000	1000
	Levels of replacements by BSFL (%)						
Fish meal	0	10.3	20.3	30.7	40.6	50.9	61.3
Fish oil	0	15.4	32.3	47.7	61.3	78.5	95.4
	Analysed chemical feed composition (g/1000 g)						
Dry matter	938.4	937.4	934.5	934.7	934.9	936.0	935.9
Crude protein	485.5	487.7	491.4	497.9	503.5	507.4	509.7
Crude fat	99.7	102.4	101	95.5	92.1	92.3	91.0
Crude fibre	6.7	9.9	13.3	16.7	19.9	23.2	26.5
Ash	82.1	82.3	82.2	80.9	81.2	80.1	80.1
Nitrogen-free extract	264.4	255.1	246.6	243.7	238.2	233.0	228.6
Ca	13.5	14.3	14.3	14.3	14.7	14.7	15.0
P	8.1	8.1	8.1	8.1	8.1	8.1	8.1
TiO_2_	3.0	2.8	2.9	3.2	3.1	3.2	3.4
Gross energy (MJ/kg)	20.39	20.44	20.44	20.43	20.42	20.42	20.43
	Calcium:phosphorus ratio (g:g)						
Ca:P ratio	1.67:1	1.77:1	1.76:1	1.76:1	1.81:1	1.81:1	1.85:1

^1^ Polfamix W, BASF Polska Ltd. Kutno, Poland; containing per 1 kg: vitamin A 1000000 IU, vitamin D3 200000 IU, vitamin E 1.5 g, vitamin K 0.2 g, vitamin B1 0.05 g, vitamin B2 0.4 g, vitamin B12 0.001 g, nicotinic acid 2.5 g, D-calcium pantothenate 1.0 g, choline chloride 7.5 g, folic acid 0.1 g, methionine 150.0 g, lysine 150.0 g, Fe 2.5 g, Mn 6.5 g, Cu 0.8 g, Co 0.04 g, Zn 4.0 g, and J 0.008 g. ^2^ Vitazol AD_3_EC, BIOWET Drwalew, Poland; containing per 1 kg: vitamin A 50 000 IU, vitamin D3 5000 IU, vitamin E 30.0 mg, vitamin C 100.0 mg. CON: control feed, H5: experimental feed with 5% BSFL, H10: experimental feed with 10% BSFL, H15: experimental feed with 15% BSFL, H20: experimental feed with 20% BSFL, H25: experimental feed with 25% BSFL, H30: experimental feed with 30% BSFL.

**Table 4:** Physical properties of experimental feeds containing black soldier fly full-fat meal.

ITEM	CON	H5	H10	H15	H20	H25	H30	SEM	*p*-Value
D (mm)	3.70 ^c^	3.72 ^b,c^	3.79 ^a,b,c^	3.85 ^a,b,c^	3.91 ^a^	3.87 ^a^	3.93 ^a^	0.02	0.0017
ER (%)	23.4 ^c^	24.1 ^b,c^	26.4 ^a,b,c^	28.4 ^a,b^	30.4 ^a^	29.0 ^a^	31.1 ^a^	0.62	0.0017
PD (g/dm^3)^	603 ^a^	603 ^a^	592 ^b^	586 ^c^	571 ^d^	546 ^e^	532 ^f^	6.21	<0.0001
SV (s/100 cm)	10.9 ^d^	10.8 ^d^	11.3 ^c,d^	11.7 ^b,c,d^	12.2 ^b,c^	12.5 ^a,b^	13.3 ^a^	0.15	<0.0010
WS (%)	97.8 ^a^	97.3 ^a^	97.4 ^a^	97.7 ^a^	96.9 ^a,b^	95.8 ^b^	96.3 ^b^	1.64	<0.0001
VI (%)	24.8 ^c^	25.2 ^c^	29.6 ^c^	36.4 ^b^	38.0 ^b^	38.4 ^b^	48.8 ^a^	0.24	<0.0001

D: pellet diameter (mm), ER: expansion ratio (%), PD: pellet density (g/dm^3^), SV: sinking velocity (s/100 cm), WS: water stability (%), VI: volume increase (%);CON: control feed, H5: experimental feed with 5% BSFL, H10: experimental feed with 10% BSFL, H15: experimental feed with 15% BSFL, H20: experimental feed with 20% BSFL, H25: experimental feed with 25% BSFL, H30: experimental feed with 30% BSFL; Different letters (a, b, c, d, e, f) indicate differences between treatments (*p* ≤ 0.05).

**Table 5:** Growth performance, feed utilization and acceptance of Siberian sturgeon fed experimental diets containing black soldier fly full-fat meal.

Parameter and Day	CON	H5	H10	H15	H20	H25	H30	SEM	*p*-Value
Growth performance parameters (Experiment 1)									
BW, day 50	83.8 ^c^	94.8 ^b^	105.7 ^a^	105.8 ^a^	106.6 ^a^	109.2 ^a^	109.1 ^a^	1.51	<0.0001
BWG, days 1–50	69.4 ^c^	80.4 ^b^	91.3 ^a^	91.4 ^a^	94.8 ^a^	92.2 ^a^	94.7 ^a^	1.49	<0.0001
SGR, days 1–50	1.91 ^c^	2.04 ^b^	2.17 ^a^	2.16 ^a^	2.20 ^a^	2.17 ^a^	2.20 ^a^	0.02	<0.0001
PWG, days 1–50	481 ^c^	558 ^b^	635 ^a^	634 ^a^	658 ^a^	641 ^a^	658 ^a^	10.0	<0.0001
Feed utilization parameters (Experiment 1)									
FI, days 1–50	61	63.3	63.6	64.3	64.6	63.3	64.25	0.50	0.2915
FCR, days1–50	0.88 ^a^	0.79 ^b^	0.69 ^c^	0.70 ^c^	0.68 ^c^	0.68 ^c^	0.68 ^c^	0.01	<0.0001
PER, days 1–50	2.34 ^c^	2.60 ^b^	2.92 ^a^	2.86 ^a^	2.91 ^a^	2.88 ^a^	2.90 ^a^	0.18	<0.0001
Feed acceptance (Experiment 2)									
FA (% of BW)	0.21 ^b^	0.21 ^b^	0.44 ^a^	0.49 ^a^	0.54 ^a^	0.62a	0.61 ^a^	0.04	0.0016

CON: control feed, H5: experimental feed with 5% BSFL, H10: experimental feed with 10% BSFL, H15: experimental feed with 15% BSFL, H20: experimental feed with 20% BSFL, H25: experimental feed with 25% BSFL, H30: experimental feed with 30% BSFL; Different letters (a, b, c) indicate differences between treatments (*p* ≤ 0.05).
